# Determination of the Optimal Level of Dietary Zinc for Newly Weaned Pigs: A Dose-Response Study

**DOI:** 10.3390/ani12121552

**Published:** 2022-06-15

**Authors:** Sally V. Hansen, Natalja P. Nørskov, Jan V. Nørgaard, Tofuko A. Woyengo, Hanne D. Poulsen, Tina S. Nielsen

**Affiliations:** Department of Animal Science, Aarhus University, Blichers Allé 20, 8830 Tjele, Denmark; sally.hansen@anis.au.dk (S.V.H.); natalja.norskov@anis.au.dk (N.P.N.); janvnoergaard@anis.au.dk (J.V.N.); woyengo@anis.au.dk (T.A.W.); hdp@anis.au.dk (H.D.P.)

**Keywords:** serum zinc, growth performance, diarrhoea, zinc oxide, intestinal integrity

## Abstract

**Simple Summary:**

Piglets have a very low feed intake immediately after weaning. We hypothesise that the EU-legislated maximum dietary zinc concentration (150 mg zinc/kg diet) will increase the risk of zinc deficiency after weaning. Zinc deficiency includes symptoms such as impaired growth and increased risk of diarrhoea. However, a high dietary zinc concentration has an antimicrobial effect on the bacteria and increases the risk of antimicrobial resistance. The findings of this study show that the dietary zinc level had a quadratic effect on growth, with a turning point at an approximately 1400 mg zinc per kg diet. The risk of diarrhoea increased up to 60% for pigs that had a blood zinc concentration which decreased after weaning. Maintaining the blood zinc concentration seven days after weaning required up to 1121 mg zinc per kg diet. There was no evidence for an antimicrobial effect when feeding pigs a diet with up to 1601 mg zinc per kg.

**Abstract:**

One hundred and eighty individually housed piglets with an initial body weight of 7.63 ± 0.98 kg (at 28 days of age) were fed a diet containing either 153, 493, 1022, 1601, 2052 or 2407 mg zinc/kg (added Zn as zinc oxide; ZnO) from day 0–21 post weaning to determine the optimal level of Zn for weaned piglets. Body weight, feed intake and faecal scores were recorded, and blood and faecal samples were collected. Dietary Zn content quadratically affected both feed intake and gain in the first two weeks, with an approximately 1400 mg Zn/kg diet and a Zn intake of 400 mg/day as the optimal levels. The relative risk of diarrhoea increased up to 60% at day 7 and 14 if serum Zn status dropped below the weaning level (767 µg/L), and maintain the weaning serum Zn status required approximately 1100 mg Zn/kg (166 mg Zn/day) during week 1. Blood markers of intestinal integrity (D-lactate and diamine oxidase) were unaffected by dietary Zn, and dietary Zn levels of 1022 and 1601 mg/kg did not affect the faecal numbers of total bacteria, Lactobacilli and E. Coli bacteria compared to 153 mg Zn/kg. These results indicate that the requirement for Zn in newly weaned piglets may be substantially higher than currently assumed.

## 1. Introduction

Zinc (Zn) is an essential micronutrient necessary for multiple structural and biological functions, including enzyme function, DNA and RNA metabolism, protein synthesis, gene expression, cell proliferation and differentiation, and cell-mediated immunity [1,2]. Thus, Zn is essential for normal growth and development. All body tissues contain Zn, but Zn stores are small and only up to approximately 15% of whole-body Zn can be mobilised during insufficient Zn intake [3,4,5]. Skin lesions or parakeratosis are well-known clinical symptoms of long-term Zn deficiency in both pigs and humans [6,7,8,9], whereas reduced feed intake may be a more short-term sign of Zn deficiency [10,11,12]. Moreover, human studies link Zn deficiency (low Zn level in blood) to diarrhoea [13,14]. Zinc deficiency impairs function of the immune system and compromises intestinal function [15,16]. This may increase the risk of entero-viral pathogen infections, triggering mal-absorption and mal-excretion of nutrients and electrolytes, thereby leading to diarrhoea [17]. However, the exact pathophysiological mechanism linking diarrhoea to Zn deficiency is yet to be elucidated. Overall, adequate daily Zn intake is essential for optimal production and health. According to the National Research Council (NRC), a Zn intake of 26.6–46.8 mg/day for piglets in the weight interval between 5 to 11 kg is recommended [18]. However, these recommendations are calculated estimates not based on results from Zn dose–response studies in weaned piglets [18]. Furthermore, the NRC recommendations on Zn supply have not been revised since 1979, despite the fact that modern pigs’ genetic potential for growth has increased substantially over the years [19]. This renders it likely that the Zn requirements have also increased since 1979.

Moreover, from June 2022, the maximal allowed dietary Zn level for weaned piglets in the EU will be 150 mg/kg diet [20,21]. With a dietary Zn level of 150 mg/kg, a newly weaned pig of 7 kg should consume 312 g feed/day to achieve the current daily recommended Zn intake of 48.6 mg/day [18]. However, several studies report that daily feed intake during the first week post-weaning (PW) is low and may range from <50 to 235 g/day [22,23,24,25]. In this feed intake range, the dietary Zn concentration should optimally be 113–936 mg/kg to achieve the NRC’s current recommended daily Zn intake. With the 150 mg Zn/kg diet, there is a chance that many low-feed-intake pigs will be undersupplied with Zn during the first period PW.

Generally, nutrient requirements can be estimated through dose–response experiments by measuring many biological endpoints, including production and biochemical parameters that the nutrient of interest affects. Thus, the adequate level of a nutrient depends on the endpoint, as some endpoints are more sensitive to deprivation of the nutrient than others [26,27]. In humans, linear growth is the only recommended functional indicator of Zn requirement, because increased growth as a result of Zn supplementation can only be interpreted as an indication of a pre-existing Zn deficiency [28]. Similar statements have been made about Zn deficiency in pigs, as it will impair growth performance [29]. Some dose–response studies have investigated the Zn requirements in weaned pigs [30,31], but these experiments included an acclimatisation period of one to two weeks, and the results can therefore not be used to estimate the Zn requirement immediately PW. Furthermore, these studies were not designed to study effects on growth and feed intake. In the current dose–response study, the aim was to determine the optimal dietary level of Zn (supplemented as zinc oxide; ZnO) for pigs the first three weeks PW, and the endpoints were feed intake, growth performance, serum Zn status, faecal scores, blood biomarkers of intestinal integrity (D-lactate and diamine oxidase activity [DAO]) and faecal microbial composition. Especially the latter was intended to illuminate when and if supra-nutritional levels of dietary Zn were supplied, since Zn-induced modifications of the gut microbial community have been used to distinguish between nutritional and pharmacological effects [29]. It was hypothesised that 150 mg Zn/kg diet is insufficient to provide newly weaned pigs with the currently recommended 46.8 mg Zn/day due to low feed intake, but also that the optimal Zn supply immediately PW may be substantially higher than what NRC recommendations currently indicate.

## 2. Materials and Methods

### 2.1. Animals, Housing, and Experimental Diet

The experiment was carried out with 180 crossbred ([Danish Landrace × Yorkshire] × Duroc) piglets (90 males and 90 females) obtained from a commercial pig herd. They arrived at the experimental facility on the day of weaning (day 0), 28 days of age (initial weight 7.63 ± 0.98 kg). Upon arrival (d 0), pigs were randomly distributed to one of six diets (*n* = 30/diet) after blocking according to body weight and gender. Pigs were housed individually in pens (1.5 × 2.4 m, 1/3 of the area slattered floor) with access to snout-contact to the neighbouring pig, ad libitum access to water and feed and provided with a 12-h light/dark cycle. The experiment was conducted in eight blocks repeated over time with 24 pigs/block (4 pigs/treatment/block) in block 1–7 and 12 pigs/block (2 pigs/treatment/block) in block 8.

The basic experimental diet was formulated to meet the Danish recommendations for nutrients for pigs between 6 and 15 kg (Table 1) but without Zn in the vitamin-mineral mixture. The grain ingredients were milled through a 3 mm screen before being combined with the remaining ingredients. High purity (80%) ZnO (VetZink, Vepidan Aps, Løgstør, Denmark) was added separately to generate six diets with increasing Zn concentration (Table 1). Diets were pelleted at 60 ℃ into 2 mm pellets and pigs received the experimental diet throughout the experiment.

### 2.2. Registrations

To calculate average daily gain (ADG) and average daily feed intake (ADFI), pigs and feed residues were weighed daily from day 0 to day 14 and again on day 21. The pigs were euthanized if they lost more than 15% of the initial weight.

The faecal score was assessed daily throughout the experiment based on a four-category scale where faecal score 1 and 2 represented normal faeces (firm and shaped to soft and shaped) and score 3 and 4 represented diarrhoea (loose to watery faeces) [32]. Pigs were immediately treated with antibiotic if their faeces were categorised as score 4. If the faeces were categorised as a score 3, the potential antibiotic treatment was postponed to the following day to await another faecal scoring.

### 2.3. Feed and Blood Sample Collection

At the beginning of each block, a representative sample of the experimental diets were obtained and at the end of the experiment, the samples were pooled for each diet and a representative sample of each diet was used for further analysis.

Blood samples from the jugular vein were obtained on day 0, 7, 14 and 21 PW. For mineral analysis, the blood samples were collected in vacutainers specifically for mineral analysis (Becton Dickinson AS, Kongens Lyngby, Denmark), and the serum was derived by centrifugation (1300× *g* for 10 min at 4 °C) and stored in polyethene tubes at −20 °C. For analysis of DAO (only day 21) and D-lactate concentration (day 0, 7, 14 and 21), the blood samples were collected in Na/Hep vacutainers, centrifuged (1300× *g* for 10 min at 4 °C) and stored in two 2 mL cryotubes at −20 °C.

### 2.4. Feed and Blood Mineral Analyses

The dry matter content of diets was determined by drying the samples at 103 °C for 20 h. The feed was analysed for Zn and copper (Cu) concentration, while serum was analysed for Zn concentration. The feed sample was ground to 1 mm and acidified with 5 mL HNO_3_ (65%), followed by destruction at 1500 W at 230 °C for 35 min using a microwave system (Ultra wave, single reaction chamber, Milestone, Shelton, CT, USA). Serum samples were prepared by adding 800 µL serum and 7200 µL 2% HNO_3_ to a 15 mL PP tube and afterwards centrifuged (16,000× *g* for 10 min at 5 °C). Hereafter, 1–2 mL of the supernatant was filtered through a 0.20 µm filter into a PP microtube and further diluted 5 times with 0.1% HNO_3_. The mineral content was measured on an iCAP TQ ICP-MS (Inductively Coupled Plasma-Mass Spectrometer) equipped with a MicroMist DC nebulizer, a Quartz cyclonic spray chamber operated at 2.7 °C (Thermo Scientific, Bremen, Germany) and a CETAC auto sampler model ASX 560. The instrument settings were forward power 1550 W, plasma gas (Ar) 14 L/min, nebulizer gas (Ar) 0.96 L/min, auxiliary gas (Ar) 0.8 L/min. The sample uptake was approximately 0.4 mL/min. Data were collected using the QtegraTM version 2.10.9.3324.131 (Thermo Fisher Scientific, Bremen, Germany). Two isotopes of Zn and Cu were measured: ^64^Zn, ^66^Zn, ^63^Cu and ^65^Cu. The ^66^Zn and ^63^Cu isotopes were used as quantifier and ^64^Zn and ^65^Cu as qualifier. Both isotopes were measured in KED mode. The standard curve contained Zn and Cu in a concentration ranging from 0.3125 to 250 ppb with ^45^Sc, ^71^Ga and ^103^Rh as calibration standards (AQ0-053-841 bought from Labsupport).

The method was validated by spiking standards of Zn and Cu to in-house reference plasma and calculating the recovery and accuracy by using five replicates per spiked concentration. The mean recoveries for ^66^Zn and ^63^Cu were 87% and 94%, respectively, with relative standard deviation between 2% and 8%.

### 2.5. Analysis of DAO and D-Lactate in Plasma

D-lactate was analysed according to Larsen [33]. Diamine oxidase (DAO) was determined by a kinetic-fluorometric method where 1,5 diamino pentane (cadaverine, Sigma C8561) was the substrate, and 10-acetyl-3,7-dihydroxyphenoxazine (ADHP) was the profluophore oxidised by the developed hydrogen peroxide. Units were defined as d-emission per min at 590 nm after excitation at 544 nm.

### 2.6. Faecal Microbial Analysis

Faecal samples obtained at day 21 from 20 randomly selected pigs receiving a 153, 1022, 1601 or 2407 mg Zn/kg diet were analysed for the number of total bacteria, total *Lactobacilli* and total *E. coli*. Extraction of DNA from 50mg of digesta samples was performed following the manufacturer’s guidelines using the Nucleospin Fecal DNA extraction kit (Machery-Nagel, Düren, Germany). Thereafter, DNA concentration was quantified using a Qubit fluorometer 3.0 (Life Technologies). Target groups were quantified from DNA-extracted samples using qPCR and a set of primers as described in Table 2 (Sigma-Aldrich). The annealing temperature, primer concentration and standard for each pair of primers are listed in Table 1. For each reaction, solution containing 5 µL of RealQ Plus 2x Master Mix, green (low ROX) (Amplicon III, Denmark), primers in concentrations as stated in Table 2, 2 µL of template DNA (template DNA was diluted ×100 for total bacteria (All bacteria) primers), and nuclease-free water up to the final volume of 10 µL were used. The qPCR analysis was performed using a MicroAmp Optical 384-well reaction plate (Applied Biosystems) and an ABI ViiA7 real-time PCR system (Thermo Fisher Scientific) under the following run conditions: pre-treatment of 2 min at 50 °C, followed by initial denaturation (15 min at 95 °C) and subsequently 40 cycles of denaturation for 15 s at 95 °C, 30 s for primer annealing at different temperatures (Table 2), and 30 s at 72 °C for base extension. Melting curves were derived by increasing the temperature from 60 to 95 °C at a rate of 0.05 °C/s, recording continuously. These curves were used to evaluate the quality of the PCR products. All analyses were performed in triplicate and a no-template control was included in every run. DNA from pure cultures was used to generate standard curves (see Table 2). Copy number in the standards was calculated from the genome size and the DNA concentration using the DNA to copy number calculator [34]. The concentrations of target DNA in the samples were estimated using PCR cycle threshold values, using QuantStudio real-time PCR software version 3.1 (Thermo Fisher Scientific).

### 2.7. Statistical Analyses

The statistical analyses of data were performed using R studio version 1.4 [38] as a randomized complete block design with the individual pig as the experimental unit. Shapiro–Wilk normality tests, Q–Q plots and residual plots verified the normality of the data. Dietary Zn level, gender and initial weight were included as fixed effect and block as random effect in all statistical analyses. Insignificant variables were omitted from the models. Linear mixed models tested the ADFI and ADG with linear and quadratic effects of dietary Zn concentration using the *lme4* package [39]. The DAO activity and D-lactate concentration was also tested with a linear mixed model which included dietary Zn level as a categorical variable and block as random effect. Lastly, linear mixed models tested also the ADG with linear and quadratic effects of serum Zn concentration, as well as the number of bacteria with the total Zn intake as a fixed effect. A generalized additive mixed model (GAMM) tested serum Zn concentration through a smooth term of dietary Zn concentration with six dimensions and the initial serum Zn at day 0 using the *mgcv* package [40]. The data were fitted using a gamma distribution and a log-transformation, and results are shown as transformed log-results. A cumulative logit mixed model with faecal score as categorical variable was tested using an ordinal logistic regression from the package *ordinal* [41]. The model included pig as a random effect, and the obtained values were square means of log odds, transformed to probabilities of transformed log odds. A cumulative logit mixed model also tested whether the daily feed intake was above or below 312 g. The model included an interaction of day and dietary Zn concentration as a fixed effect and pig as random effect. The relative risk of diarrhoea in relation to serum Zn status above or below the serum Zn level at weaning was evaluated with the package *fmsb* [42]. Values are presented as LS-means with 95% confidence interval (95% CI), obtained with the *emmeans* package [43]. A threshold of *p* ≤ 0.05 was considered as statistically significant while *p*-Values between <0.05 and ≤0.10 were considered as a statistical tendency.

## 3. Results

### 3.1. Feed, Feed Intake and Weight Gain

One hundred and seventy-nine pigs completed the experiment, as one pig was euthanized as it lost more than 15% of its initial weight. The basal diet had a higher Zn content than expected (50 vs. 29 mg/kg), but the experimental diets showed a Zn content close to the intended levels (Table 1). The deviation between calculated and analysed Zn concentration was 2–7% in the six dietary groups.

**Figure 1:**
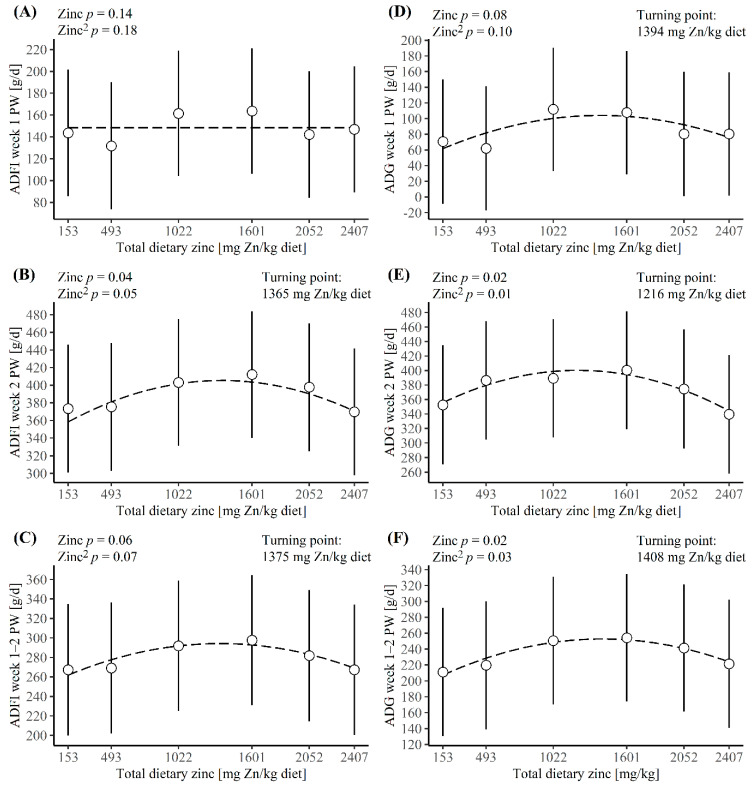
The average daily feed intake (ADFI) and average daily gain (ADG) obtained with six dietary zinc concentration during (**A**,**D**) the first week post-weaning (PW), (**B**,**E**) the second week PW and (**C**,**F**) the two first weeks PW. The dashed lines illustrate the effect of dietary zinc concentration on ADFI or ADG. The turning point and *p*-value of the linear and quadratic parameters are denoted at the top of each graph.

Dietary Zn concentration did not affect the ADFI during the first week PW (*p* ≥ 0.14, Figure 1A) and there was a very high probability (0.84–1.00) for pigs eating less than 312 g/day during day 2–7 PW (Figure S1). Even though dietary Zn level had no effect on the feed intake, it had an effect on the Zn intake during the first week PW, as pigs fed a diet with 153 mg dietary Zn/kg showed the lowest Zn intake of less than 23 mg/day (*p* < 0.05, Figure S2). ADFI had a strong tendency of being quadratic, affected by dietary Zn concentration during the second week PW (*p* = 0.05, Figure 1B) and the first two weeks PW together (*p* = 0. 07, Figure 1C), while overall (week 1–3 PW), the effect was significant (*p* = 0.04, data not shown). The estimated turning points occurred at dietary Zn levels of 1364 mg/kg during the second week PW, 1375 mg/kg during the first two weeks PW and 1344 mg/kg overall. These dietary Zn levels would result in ADFI of 405 g/day, 287 g/day and 433 g/day, respectively (Table S1). These dietary Zn levels were estimated to a Zn intake of 553 mg/day during the second week PW, 395 mg/day during the first two weeks PW and 583 mg/day during the three weeks PW.

Dietary Zn concentration showed a tendency for a quadratic effect on the ADG during the first week PW (*p* = 0.10, Figure 1D), and a significant quadratic effect during the second week PW (*p* = 0.01, Figure 1E), the first two weeks PW (*p* = 0.03, Figure 1F) and overall (Week 1–3 PW, *p < 0.01*, data not shown). The estimated turning points occurred at dietary Zn levels of 1394 mg/kg for the first week PW, 1216 mg/kg for the second week PW and 1408 mg/kg for the first two weeks PW. These dietary Zn levels would result in ADG of 104 g/day, 401 g/day and 253 g/day, respectively (Table S2). These dietary Zn levels would lead to an estimated Zn intake of 207 mg/day during the first week PW, 492 mg/day during the second week PW and 404 mg/day during the first two weeks PW. The overall ADG during the three weeks PW was estimated to have a turning point at a dietary Zn level of 1265 mg/kg with an ADG of 367 g/day, and this dietary Zn level would lead to a Zn intake of 547 mg/day.

### 3.2. Diarrhoea Probability

The probability of diarrhoea (faecal score ≥ 3 on a scale from 1 to 4) was higher with the 153 mg Zn/kg diet compared to 2407 mg Zn/kg at any time point (Table 3). Moreover, from the second week, the 2052 mg Zn/kg diet had a lower diarrhoea probability than the 153 mg Zn/kg diet (*p* = 0.03). Diarrhoea probability was similar for the dietary Zn levels between 153 and 1601 mg/kg at any time point (*p* > 0.66), and dietary Zn levels between 493 and 1601 mg Zn/kg diet had a similar diarrhoea probability as 2052 mg/kg at any time point (*p* > 0.29). However, in the third week, the 153 mg Zn/kg diet tended to show a higher probability than 1601 mg Zn/kg (*p* = 0.09). The diarrhoea probability for week 1–2 had a tendency of being higher for the 493 mg Zn/kg diet compared to the 2407 mg Zn/kg diet (*p* = 0.09).

### 3.3. Serum Zn Status

The initial serum Zn concentration at weaning was similar for the six dietary treatment groups (767 ± 19 µg/L, *p* > 0.05, data not shown). Pigs with a serum Zn level lower than 767 µg/L at day 7 PW had a 52% higher risk of a diarrheal episode during the second week PW compared to pigs with a serum Zn level higher than 767 µg/L. The risk of a diarrheal episode increased to 60% in the third week PW for pigs with a serum Zn level at day 14 lower than 767 µg/L (Table 4).

**Figure 2:**
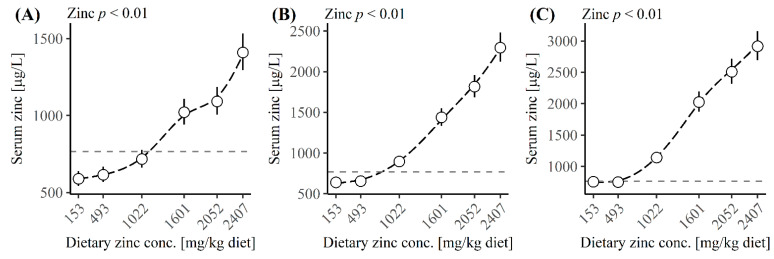
Average serum zinc concentration [μg/L] at (**A**) day 7 post-weaning (PW), (**B**) day 14 PW, (**C**) day 21 PW. The black dashed lines illustrate the effect of dietary zinc concentration, while the grey dashed line illustrates the serum zinc concentration at day 0.

Figure 2 shows the relation between serum Zn concentration and the dietary Zn concentration at day 7, 14 and 21 PW (*p* < 0.01). Maintaining the serum Zn level at 767 µg/L at day 7 PW required the dietary Zn concentration to be 1121 mg/kg (Figure 2A), which would lead to a Zn intake of 166 mg/day during the first week PW. The dietary Zn concentration required to maintain the serum Zn level at 767 µg/L at day 14 and 21 PW decreased to 778 and 461 mg/kg, respectively (Figure 2B,C), which would lead to a Zn intake of 307 and 209 mg/day tin he second and third week PW.

### 3.4. Serum Zn Status and Weight Gain

**Figure 3:**
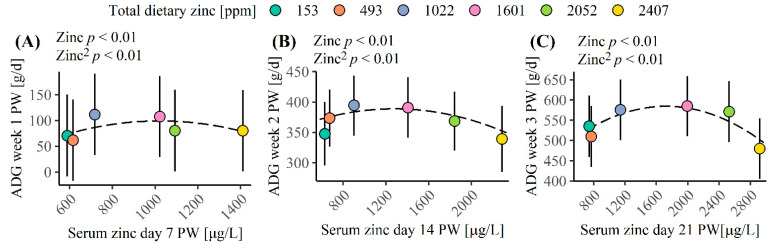
Average daily gain (ADG) as a function of serum zinc concentration. (**A**) The ADG for the first week post-weaning (PW) and the mean serum zinc concentrations at day 7 PW. (**B**) The ADG for the second week PW and the mean serum zinc concentrations at day 14 PW. (**C**) The ADG for the third week PW and the mean serum zinc concentrations at day 21 PW (30 pigs/diet). The black dashed line illustrates the effect of serum zinc concentration on ADG.

Figure 3 shows the quadratic correlation between serum Zn concentration and the ADG. The highest ADG during the first week PW was observed with a serum Zn level of 717 µg Zn/L (dietary Zn concentration of 1022 mg Zn/kg, Figure 3A), but the turning point was calculated to be 1011 µg Zn/L serum (Table S3). The highest ADG during the second week PW was obtained with 1022–1601 mg Zn/kg in the diet, which had a serum Zn concentration at day 14 PW of 902–1406 µg/L (*p* < 0.01, Figure 3B). The maximal ADG during the second week PW was estimated to occur with a serum Zn level at day 14 PW of 1279 µg/L (ADG of 389 g/day, Table S3). Obtaining this serum Zn level at day 14 PW would require 1471 mg dietary Zn/kg during the second week PW (Figure 2B). The highest ADG during the third week PW was obtained with a serum Zn concentration at day 21 PW between 1142 and 2527 µg/L (*p* < 0.01, Figure 3C). The maximal ADG during the third week PW was estimated to occur with a serum Zn concentration at day 21 PW of 1724 µg/L (ADG of 585 g/day, Table S3). Obtaining this serum Zn level at day 21 PW would require 1418 mg dietary Zn/kg during the third week PW (Figure 2C).

### 3.5. Intestinal Integrity and Faecal Bacteria

Dietary Zn level had no effect on the concentration of D-lactate at day 7, 14 and 21 PW, nor on the DAO activity at day 21 PW (*p* > 0.05, Table 5).

**Figure 4:**
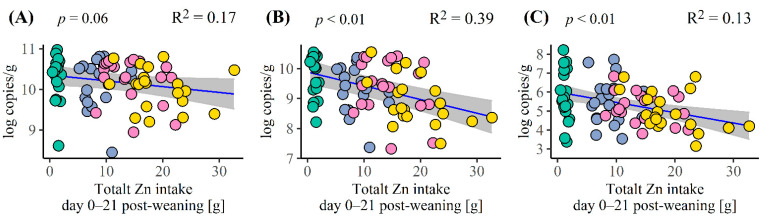
The correlation between the total zinc intake during the 21 days of the experiment and the faecal number of (**A**) total bacteria, (**B**) total *Lactobacilli* and (**C**) total *Escherichia coli* at day 21 post-weaning. The correlation is assessed based on the R^2^. The blue line illustrates the correlation and the grey area illustrate the 95% confidence interval (20 pigs/diet).

The dietary Zn concentration had a tendency of reducing the number of total faecal bacteria at day 21 (*p* = 0.08), while the number of total *E. coli* and *Lactobacilli* was reduced by the dietary Zn concentration (*p* = 0.04 and <0.01, respectively; Table 6). A dietary Zn concentration of 2407 mg/kg tended to yield a lower number of total bacteria and total *E. coli* compared to 153 mg/kg (*p* = 0.07 and 0.05, respectively; Table 6), while the number of total *Lactobacilli* was lower with 2407 mg/kg than 153 mg/kg (*p* < 0.01). Dietary Zn levels of 1022 and 1601 mg/kg yielded similar number of total bacteria, *E. coli* and *Lactobacilli* as both 153 and 2407 mg/kg (Table 6). Figure 4 shows that the numbers of total bacteria (*p* = 0.06), *Lactobacilli* (*p* < 0.01) and *E. coli* (*p* < 0.01) were negatively correlated with the total Zn intake, with R^2^ values between 0.13 and 0.39.

## 4. Discussion

The Zn requirement in weaned pigs has been investigated in several studies in the past [30,31,44,45,46]. However, the experimental setup in most of these studies included an adjustment period during the first 7–14 days PW [30,31,45] and thereby, the results are not applicable to estimating the Zn requirement in pigs during the first week PW.

### 4.1. Serum Zn Status

The risk of diarrhoea has been related to the Zn status in blood in humans with Zn deficiency [13]. Bahl et al. [13] found that children’s risk of diarrhoea increased by 47% if the plasma Zn concentration was lower than 550 μg/L compared to a higher plasma Zn level. This corresponds to our result that, if the serum Zn level decreased below the average at weaning, the risk of diarrhoea increased to 52–60%. Therefore, it seems essential to maintain the weaning serum Zn level PW if an increased risk of diarrhoea in the following weeks should be avoided. Pigs receiving a 153 to 493 mg Zn/kg diet showed reduced serum Zn status relative to the day of weaning on day 7 and 14 PW, corresponding to findings by Carlson et al. [47] and Burrough et al. [48]. We calculated that maintaining the serum Zn level at weaning required a 1121, 778 and 461 mg Zn/kg diet at day 7, 14 and 21 PW, respectively.

### 4.2. Feed Intake and Weight Gain

The calculated turning points for maximal feed intake of approximately 1400 mg/kg at week 2 and week 1–2 is in agreement with the results of Hill et al. [46]. They reported a quadratic effect of dietary Zn content on ADFI and the highest ADFI was achieved when pigs were fed a diet with a 1500 mg Zn/kg diet supplementation, compared to 0 or 3000 mg Zn/kg diets. Since many studies apply only two dietary Zn concentrations, low and a high (typically 100–250 and 2000–3000 mg Zn/kg diet), this may explain why some studies report no effect of dietary Zn content on the feed intake, as our results show that the ADFI was similar for 153 and >2000 mg Zn/kg diets [49,50,51]. The quadratic effect of dietary Zn content on feed intake may relate to the influence of Zn on the palatability of feed. Reynolds et al. [52] found that, when pigs weaned at day 28 were given the choice between a Zn unsupplemented diet and a diet supplemented with 3100 mg Zn/kg (as ZnO), up to 91 % of the total feed consumption was of the unsupplemented diet. Zhang and Guo [53] and Yin et al. [54] found a higher concentration of circulating ghrelin in pigs (24–28 days of age) fed a 2000 mg Zn/kg diet compared to a 100–130 mg Zn/kg diet. Ghrelin secreted from enteroendocrine cells of the gastrointestinal tract is involved in appetite regulation, and increased ghrelin secretion stimulates appetite [55]. It can therefore be speculated that the highest feed intake, which we calculated to coincide with approximately a 1400 mg Zn/kg diet, represents a balance between increased ghrelin secretion with higher levels of dietary Zn and reduced palatability of feed with high levels of ZnO.

In Poulsen [44], six dietary Zn supplementation levels (0, 100, 200, 1000, 2500 and 4000 mg/kg diet) were provided from weaning at 28 days of age and for the following five weeks. The ADG decreased when the dietary Zn supplementation was above 2500 mg/kg. However, as there were no supplementation doses between 1000 and 2500 mg Zn/kg, it is unknown what the ADG would have been in this range. Hahn and Baker [45] included no adjustment period PW (weaning at day 28 of age), and showed similar growth performance following six Zn supplementation doses (0, 250, 500, 1000, 3000 or 5000 mg/kg diet). Antibiotics were added to all diets in this study, and therefore, results cannot be compared with our results. Some studies have shown that a 2000–3000 mg Zn/kg diet increase the ADG of weaned pigs compared to a 100–150 mg Zn/kg diet during the first four weeks PW [45,49,56]. However, the quadratic effect of dietary Zn on ADG shown in the present study corresponds to other studies [44,46,57]. A quadratic effect of dietary Zn level on ADG may explain why other studies observed no effect of dietary Zn content on ADG, as it has been most common to compare a <250 mg Zn/kg diet to a >2000 mg Zn/kg diet [22,51,58]. Kjeldsen et al. [59] showed in a large Danish farm trial (750 pigs/diet) that a dietary Zn level of 2500 mg/kg compared to 1500 mg/kg resulted in a slightly higher feed intake (264 g/day vs. 247 g/day) and ADG (222 g/day vs. 207 g/day) during the first 11 days PW. From day 14 to 30 kg, all pigs were fed a diet with 100 mg added Zn/kg diet, and over the entire experimental period (7–30 kg) there was no difference in production parameters (gain, feed intake, feed conversion ratio) between the 1500 and 2500 mg Zn/kg diet. Pigs fed only a 100 mg Zn/kg diet during the entire experimental period showed reduced production results, indicating that adding 100 mg Zn/kg diet is not enough to fulfil the Zn requirement. The optimal dietary Zn concentration for the feed intake and growth of individually housed, newly weaned pigs was estimated to be approximately 1400 mg Zn/kg, but the requirement might differ for commercial pigs due to higher feed intake for group-housed pigs, lower sanitary levels, heat stress, etc. [60,61,62,63].

The level of feed intake during the first week PW was similar to levels reported by Bruininx et al. [24,25]. After June 2022, only a 150 mg Zn/kg diet will be allowed for weaned pigs in the EU, and if the NRC’s current recommendation of 48.6 mg Zn/day for 7–11 kg pigs is then to be achieved, they should eat 312 g/day. The probability of a feed intake less than 312 g/day was more than 0.84 in the first seven days PW, and pigs fed a 153 mg Zn/kg diet had a Zn intake of less than 23 mg/day during the first seven days PW. Brugger et al. [30] estimated that pigs fed a Zn-deficient diet, which they estimated to be a dietary level less than 58 mg/kg, would reach clinical Zn deficiency after approximately 10 days due to the depletion of whole-body Zn storage. Clinical symptoms of Zn deficiency include depressed growth, reduced feed intake, diarrhoea and impaired immune function, while subclinical Zn deficiency is associated with reduced Zn status parameters such as serum Zn concentration [1]. Brugger et al. [30] mainly focused on the Zn status parameters to determine sufficient dietary Zn content, but King et al. [28] stated that increased growth (weight gain and linear growth) as a result of Zn supplementation in humans reflects Zn deficiency, and thus growth is a functional indicator of Zn requirement. If the same applies to pigs, our data indicate that the daily dietary Zn requirement for the first two weeks PW might be up to nine times higher than the NRC recommendation (48.6 mg/day), as we found the highest ADFI and ADG would lead to an average Zn intake of 395–436 mg/day.

Only Hahn and Baker [45] have previously studied the correlation between Zn status in the blood and ADG in weaned pigs. They reported a highest ADG (day 0–21) with a plasma Zn status (day 21) of approximately 1500 µg/L, which corresponds to our results. Together, these results agree well with the assumed upper limit of the adequate Zn level stated by Puls [64].

### 4.3. Intestinal Integrity

The activity of DAO and D-lactate concentration are well-established biomarkers of the integrity of the mucosal barrier in the small and large intestine, respectively [65,66], as the plasma concentration will increase if the permeability of the intestinal wall is compromised [67,68]. Some studies have shown that a high dietary Zn level (2200–3000 mg Zn/kg diet) reduces the DAO activity in plasma/serum compared to low dietary Zn levels (100 mg Zn/kg diet) [69,70]. This is in contrast to our results, but it could be due to differences in when measurements were taken PW (7–14 vs. 21 days), as well as the methods used to determine the level of activity. However, the lack of an effect of dietary Zn on D-lactate corresponds to findings from other studies [70,71,72]. Overall, these results indicate that the integrity of the intestines is unaffected by the level of dietary Zn.

### 4.4. Diarrhoea Probability and Faecal Microbial Composition

Dietary Zn level affected the probability of diarrhetic faecal scores in week 1 PW, with 153 and 1601 mg Zn/kg diets having the highest probability of diarrhoea compared to the 2407 mg Zn/kg diet, which contradicts results from other studies [73,74]. However, in the second and third week PW, only pigs receiving the 153 mg Zn/kg diet had a higher probability of diarrhetic faecal scores than for the 2052 and 2407 mg Zn/kg diet. Human studies have linked Zn deficiency in children to diarrhoea [13,14,75]. It can therefore be speculated that a dietary Zn level of 153 is a sub-optimal level. The two highest dietary Zn levels showed the lowest probability of diarrhetic faecal scores, whereas the intermediate Zn concentrations were not different from either the 153 or the 2052 mg Zn/kg diet in the periods following the first week PW. Kjeldsen et al. [59] showed that there was no further beneficial effect of moving from a 1500 to a 2500 mg Zn/kg diet when evaluating the proportion of pigs receiving antibiotic treatment due to diarrhoea.

One of the arguments for no longer allowing the use of high doses of Zn for newly weaned piglets is that some studies indicate that it increases the risk of the development of antimicrobial resistance [76,77] and general anti-microbial effects on the microbiota richness [51,78]. Our results showed similar numbers of total bacteria, Lactobacilli and total coliform bacteria in faeces at dietary Zn contents of 153, 1022 and 1601 mg/kg, which is similar to the results obtained by Pieper et al. [79]. Moreover, the total Zn intake can only determine 13–17% of the variation in log copies of total bacteria and total *Lactobacilli*. Therefore, there are no indications that a 1400 mg Zn/kg diet would modulate the faecal proportions of these two groups of bacteria investigated here, or that it would exert anti-microbial effects by reducing total bacteria. However, these results cannot determine the risk of antimicrobial resistance.

## 5. Conclusions

In conclusion, more than 80% of pigs had a feed intake below 312 g/d in the first week PW, which is required for a 7 kg pig to have a Zn intake fulfilling the current NRC recommendations if the diet contains 150 mg Zn/kg. The optimal level of dietary Zn in the first two weeks PW was approximately 1400 mg/kg, corresponding to a daily intake of 400 mg Zn when ZnO is the source of added Zn and feed intake as well as growth are the primary endpoints. This is approximately nine times higher than the current NRC recommendations. There were no indications that a dietary level of a 1400 mg Zn/kg diet exceeded the nutritional level in the first two weeks PW. If the Zn status drops below the weaning level, the risk of diarrhoea increases up to 60% for the following two weeks.

## Figures and Tables

**Table 1:** Ingredients and calculated composition of the basal diet.

Ingredients	%
Wheat	40.3
Barley	20.0
Soy protein concentrate, HP300	9.8
Soybean meal, 45.8% protein	8.0
Oats	5.9
Vegetable fat and oil	4.8
Lactose	4.1
Fishmeal	3.0
Vitamin/mineral premix ^2^	2.2
Monocalcium phosphate	1.1
Salt	0.7
Aroma	0.1
Natuphos 10000 E ^1^	0.02
Calculated composition (as-fed)	
Crude protein, %	18.3
Lysine, %	1.25
Ca, %	0.74
P, % (available)	0.40
Fe, mg/kg	180
Zn, mg/kg	29
Cu, mg/kg	120
Added Zn (mg/kg) ^3^	Analysed Zn (as-fed), mg/kg feed
Basal diet (no added ZnO)	50
100	153
450	493
950	1022
1450	1601
1950	2052
2450	2407

^1^ 200% phytase = 1000 FUT/kg, Natuphos 10000 E. ^2^ Zn-free vitamin-trace mineral mix providing the following per kilogram of diet: 5989 IU vitamin A, 598 IU vitamin D3, 156 mg Vitamin E, 2.4 mg vitamin K3, 2.4 mg vitamin B1, 4.8 mg vitamin B2, 3.6 mg vitamin B6, 0.02 mg vitamin B12, 24 mg Niacin, 0.24 mg Biotin, 12 mg pantothenic acid, 6.0 mg Ca, 6.0 mg P, 3.3 mg Na, 5.1 mg Kl, 180 mg Fe (FeSO4), 120 mg Cu (CuSO4), 48 mg Mn (MnO), 0.2 mg Se (Na2SeO3). ^3^ High purity ZnO (80%) VetZink, Vepidan Aps, Løgstør, Denmark.

**Table 2:** Primers and quantitative PCR conditions used for real-time PCR.

Primer Name ^1^	Target Sequence	Sequence (5′–3′)	Conc. ^2^ (μM)	AT ^3^ (°C)	Size (bp)	Standard	Reference
Bank-lacto-F	All *Lactobacillus* (23S rRNA)	GCGGTGAAATTCCAAACG	0.30	60	216	*Lactobacillus reuteri* DSM 20016	[35]
Bank-lacto-R		GGGACCTTAACTGGTGAT	0.30				
*E. coli* 401 F	All *E. coli* (ybbW gene)	TGATTGGCAAAATCTGGCCG	0.50	65	211	*E. coli* K12	[36]
*E. coli* 611 R		GAAATCGCCCAAATCGCCAT	0.50				
16S_BAC-F (SRV3-1)	All bacteria (16S rRNA)	CGGYCCAGACTCCTACGG	0.30	65	200	*E. coli* K12	[37]
16S_BAC-R (SRV3-2)		TTACCGCGGCTGCTGGCAC	0.30				

^1^ F = forward; R = reverse. ^2^ Primer concentration. ^3^ AT = Annealing temperature.

**Table 3:** Probability (%) of diarrhoea in pigs in different intervals post-weaning dependent on total dietary zinc concentration ^1^.

	Total Dietary Zinc Concentration [mg Zn/kg Diet] ^2^							
	153	493	1022	1601	2052	2407	95% CI	*p*-Values
Week 1	28.2 ^a^	19.1 ^ab^	13.2 ^ab^	23.3 ^a^	10.6 ^ab^	6.6 ^b^	11.7–19.9	<0.05
Week 2	22.7 ^a^	13.6 ^ab^	16.1 ^ab^	16.0 ^ab^	6.9 ^b^	6.4 ^b^	9.6–16.0	<0.05
Week 3	43.7 ^a^	26.9 ^ab^	27.7 ^ab^	16.9 ^abc^	12.4 ^bc^	4.5 ^c^	13.1–26.0	<0.05
Week 1–2	26.3 ^a^	17.1 ^abc^	15.7 ^abc^	20.0 ^ab^	9.7 ^bc^	7.0 ^c^	12.2–17.9	<0.05
Week 1–3	33.3 ^a^	22.0 ^ab^	21.5 ^ab^	21.1 ^ab^	13.1 ^bc^	7.7 ^c^	15.5–21.6	<0.05

^1^ Probability is calculated as Prob = (odds/(1 + odds)) * 100, where odds = e^loge(odds)^. ^2^ Values are presented as least squares means in per cent. ^a, b, c^ Different letters indicate significant difference (*p* < 0.05) within the row.

**Table 4:** Relative risk of diarrheal episode over the second and third week post weaning by dividing the pigs into two groups depending on if their serum zinc level was higher or lower than weaning level (767 µg/L).

	Serum Zinc Concentration		Relative Risk (95% CI)	*p*-Value
	≤767 µg/L	>767 µg/L		
**Day 7 ^1^**				
Number of pigs	87	85		
Days with diarrhoea (d 7–13) *	126	81	1.52 (1.18–1.96)	<0.01
Total number of days ^#^	609	595		
**Day 14 ^2^**				
Number of pigs	54	111		
Days with diarrhoea (d 14–20) *	142	182	1.60 (1.34–1.92)	<0.01
Total number of days ^#^	378	777		

^1^ Serum zinc level at day 7 post-weaning. ^2^ Serum zinc level at day 14 post-weaning. * Total number of days with diarrhoea observations (faecal score 3 or 4). ^#^ Total number of days = number of pigs * 7 days.

**Table 5:** Effect of dietary Zn level on D-lactate concentration and diamine oxidase (DAO) activity in plasma.

	Dietary Zn [mg Zn/kg Diet]							
	153	493	1022	1601	2052	2407	95% CI	*p*-Value
**D-Lactate [mg/L]**								
Day 0	0.71	0.91	1.48	1.18	0.88	1.54	0.63–1.82	0.06
Day 7	2.27	3.01	2.74	2.23	1.61	1.87	1.54–3.25	0.61
Day 14	1.97	1.60	1.93	2.43	2.11	1.67	1.54–2.42	0.81
Day 21	2.10	1.11	1.43	4.14	1.48	1.03	0.92–1.96	0.47
**DAO [Units/min]**								
Day 21	100	119	104	123	123	124	82–149	0.25

**Table 6:** The number of total bacteria, total *Lactobacilli* and total *E. coli* in faeces at day 21 post-weaning (log copies/g sample, N = 20 pigs/dietary group).

	Total Dietary Zn [mg Zn/kg Diet]					
	153	1022	1601	2407	95% CI	*p*-Value
Total bacteria count (log copies/g sample)	10.4 ^A^	10.3 ^AB^	10.2 ^AB^	10.0 ^B^	10.0–10.4	0.08
Total *Lactobacilli* count (log copies/g sample)	9.80 ^a^	9.44 ^ab^	9.39 ^ab^	8.92 ^b^	8.98–9.82	<0.01
Total *E. coli* count (log copies/g sample)	5.92 ^a^	5.53 ^ab^	5.23 ^ab^	4.99 ^b^	5.08–5.73	0.04

^a,b^ Different letters indicate significant difference (*p* < 0.05). ^A,B^ Different letters indicate tendency of a difference (0.05 ≤ *p* < 0.01).

## Data Availability

Data can be obtained from the corresponding author upon reasonable request.

## Supplementary Materials

The following supporting information can be downloaded at: https://www.mdpi.com/article/10.3390/ani12121552/s1, Figure S1: The probability of pigs eating less than 312 g/day the first 14 days post-weaning. Figure S2: The average daily zinc intake during day 2–7 post-weaning. Letters indicate a significant (*p* < 0.05) difference between dietary groups within each day. Values are LS-means ± 95% CI. Table S1: Estimates of the parameters of the equation of the average daily feed intake fitted on the dietary zinc concentration. Table S2: Overview of the average daily gain equations fitted on the dietary zinc concentration. Table S3: Overview of the average daily gain equations fitted on the serum zinc concentration.
