# Supplementary material for: Determination of the Optimal Level of Dietary Zinc for Newly Weaned Pigs: A Dose-Response Study
Source: Animals (Basel). 2022 Jun 15;12(12):1552. doi: 10.3390/ani12121552 (PMC9219510; doi:10.3390/ani12121552)
Supplement: Supplementary file 1 [file animals-12-01552-s001.zip › animals-1750588-supplementary.pdf]

Supplementary Materials

Table S1. Estimates of the parameters of the equation of the average daily feed intake fitted on the dietary zinc concentration, and estimation of the dietary zinc level leading to maximal average daily feed intake.

	**Week 2 post-weaning**			**Week 1-2 post-weaning**		
**Parameter**	**Coefficients**	**SE**	***P*-value**	**Coefficients**	**SE**	***P*-value**
Zinc	0.09	0.04	0.041	0.07	0.04	0.055
$Zinc^2$	-3.2×10^{-5}	1.6×10^{-5}	0.051	-2.5×10^{-5}	1.4×10^{-5}	0.0668
Body Weight d0	45.3	3.92	<0.001	31.4	3.54	<0.001
Observations	177			176		
Marginal R^2 / Conditional R^2	0.124 / 0.354			0.085 / 0.374		

	**Turning point calculation:** [Zinc / (2 * $Zinc^2$)]	**ADFI at turning point**
Week 2 post-weaning	0.09 / (2 * -3.2×10^{-5}) ≈ 1,365 mg Zn/kg diet	0.09*1,365-3.2×10^{-5}*1,365 ^{2}+ 45.3*7.63 ≈ 405 g/d
Week 1-2 post-weaning	0.07 / (2 * -2.5×10^{-5}) ≈ 1,375 mg Zn/kg diet	0.07*1,375-2.5×10^{-5}*1,375 ^{2}+ 31.4*7.63 ≈ 287 g/d

Table S2. Estimates of the parameters of the equation of the average daily gain fitted on the dietary zinc concentration, and estimation of the dietary zinc level leading to maximal average daily gain.

	Week 1 post-weaning			Week 2 post-weaning			Week 1-2 post-weaning		
**Parameter**	**Coef.**	**SE**	***P*-value**	**Coef.**	**SE**	***P*-value**	**Coef.**	**SE**	***P*-value**
Intercept	50.6	35.0	0.166	69.4	86.7	0.425	41.6	74.0	0.575
Zinc	0.08	0.04	0.082	0.10	0.04	0.030	0.08	0.04	0.028
$Zinc^2$	-2.8×10^{-5}	1.7×10^{-5}	0.102	-4.0×10^{-5}	1.7×10^{-5}	0.019	-2.9×10^{-5}	1.4×10^{-5}	0.042
Body Weight d0	-	-	-	35.8	10.6	0.001	20.2	8.80	0.023
Observations	178			178			177		
Marginal R^2 / Conditional R^2	0.126 / 0.348			0.126 / 0.348			0.061 / 0.430		

	**Turning point calculation:** [Zinc / (2 * $Zinc^2$)]	**ADG at turning point**
Week 1 post-weaning	$0.08 / (2 * 2.8\times10^{-5}) \approx 1{,}394$ mg Zn/kg diet	$50.6+0.08*1{,}394-2.8\times10^{-5}*1{,}384^{2} \approx 104$ g/d
Week 2 post-weaning	$0.10 / (2 * -4.0\times10^{-5}) \approx 1{,}216$ mg Zn/kg diet	$69.4+0.10*1{,}216-1.7\times10^{-5}*1{,}216^{2}+ 35.8*7.63 \approx 401$ g/d
Week 1-2 post-weaning	$0.08 / (2 * -2.9\times10^{-5}) \approx 1{,}408$ mg Zn/kg diet	$41.6+0.08*1{,}408-2.9\times10^{-5}*1{,}408^{2}+ 20.2*7.63 \approx 253$ g/d

Table S3. Estimates of the parameters of the equation of the average daily gain fitted on the serum zinc concentration, and estimation of the serum zinc level leading to maximal average daily gain.

	**Week 1 post-weaning**			**Week 2 post-weaning**			**Week 3 post-weaning**		
**Parameters**	**Coef.**	**SE**	***P*-value**	**Coef.**	**SE**	***P*-value**	**Coef.**	**SE**	***P*-value**
Intercept	-43.4	39.4	0.578	330.2	28.7	<0.001	411.2	29.7	<0.001
Serum zinc*	0.28	0.06	<0.001	0.09	0.02	<0.001	0.20	0.02	<0.001
Serum zinc^2	-1.4×10^{-4}	3.2×10^{-5}	<0.001	-3.6×10^{-5}	6.0×10^{-5}	<0.001	-5.8×10^{-5}	5.0×10^{-5}	<0.001
Observations	48			48			48		
Marginal R^2 / Conditional R^2	0.011 / 0.963			0.038 / 0.963			0.111 / 0.962		

	**Turning point calculation:** [SZinc / (2 * SZinc^2)]	**ADG at turning point**
Week 1 post-weaning	$0.28 / (2 * -1.4\times10^{-4}) \approx 1{,}012$ μg/L	$-3.4+0.28*1{,}012-1.4\times10^{-4}*1{,}012^2 \approx 99$ g/d
Week 2 post-weaning	$0.09 / (2 * -3.6\times10^{-5}) \approx 1{,}279$ μg/L	$330.2+0.09*1{,}279-3.6\times10^{-5}*1{,}279^2 \approx 389$ g/d
Week 3 post-weaning	$0.20 / (2 * -5.8\times10^{-5}) \approx 1{,}724$ μg/L	$411.2+0.20*1{,}724-5.8\times10^{-5}*1{,}724^2 \approx 585$g/d

* Serum zinc concentration at the end of each week (Week 1 = Day 7, Week 2 = Day 14, Week 3 = Day 21)

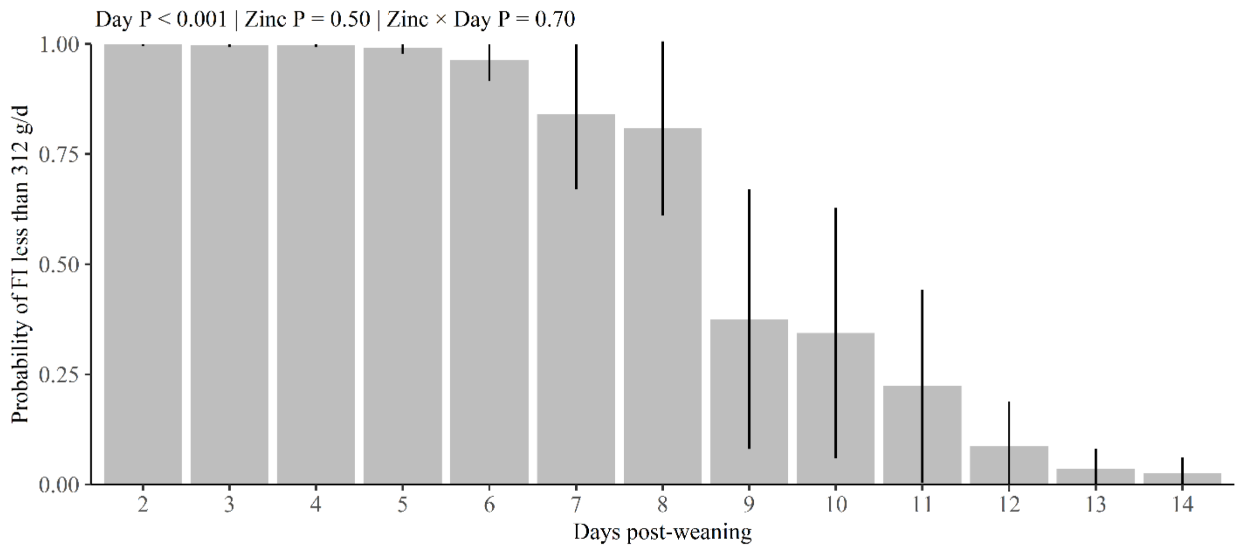

Figure S1. The probability of pigs eating less than 312 g/day the first 14 days post-weaning. Values are LS-means ± 95% CI, 30 pigs/diet.

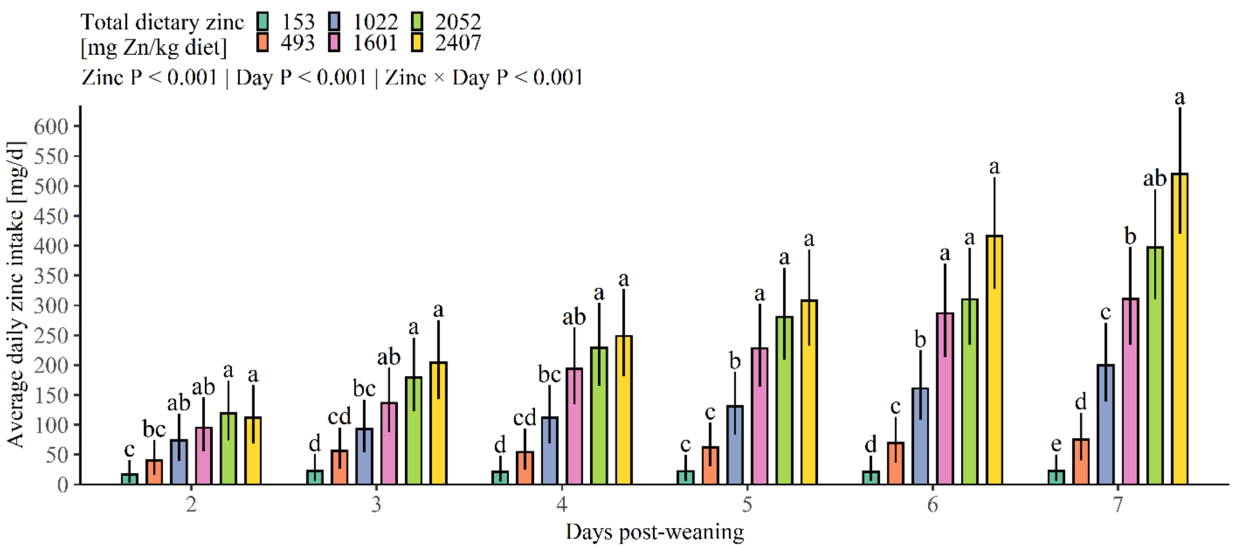

Figure S2. The average daily zinc intake during day 2-7 post-weaning. Letters indicate a significant (P < 0.05) difference between dietary groups within each day. Values are LS-means ± 95% CI, 30 pigs/diet.
